# Validating self-reported strokes in a longitudinal UK cohort study (Whitehall II): Extracting information from hospital medical records versus the Hospital Episode Statistics database

**DOI:** 10.1186/1471-2288-12-83

**Published:** 2012-06-21

**Authors:** Annie Britton, Beverly Milne, Therese Butler, Adelaida Sanchez-Galvez, Martin Shipley, Anthony Rudd, Charles DA Wolfe, Ajay Bhalla, Eric J Brunner

**Affiliations:** 1 Department of Epidemiology and Public Health, University College London, 1-19 Torrington Place, London, WC1E 6BT, UK; 2 Division of Health and Social Care Research, King’s College London, 7th Floor Capital House, 42 Weston Street, London, SE1 3QD, UK

**Keywords:** Stroke, Cohort studies, Self-report, Validation, Medical records, NHS HES database

## Abstract

**Background:**

Valuable information on the determinants of non-fatal stroke can be obtained from longitudinal observational cohort studies. Such studies often rely on self-reported stroke events, which are best validated with external medical evidence. The aim of this paper is to compare the information on incident non-fatal stroke events arising from different sources.

**Methods:**

We carried out a validation of self-reported stoke events among participants in the Whitehall II Study, a large UK based cohort study (baseline sample size 10,308 men and women).

**Results:**

106 stroke events were self-reported in three self-administered questionnaires between 2002 and 2009. Eight (7.5%) of these events were discarded as false positives after medical review, 66 were validated by information from the NHS Hospital Episode Statistics (HES) database in England, 16 by manual searches of hospital records alone, and 12 by letters from general practitioners alone. HES provided information on an additional (i.e. not self-reported) 47 events coded as stroke during the period 2002 to 2009 in hospitals in England among the original baseline participants. Of these, 43 participants were no longer active in the study and 4 had completed questionnaires but not reported a stroke event.

**Conclusions:**

Validating self-reported strokes in cohort studies with information from the NHS HES database was efficient and provided information on probable non-fatal stroke events among cohort members no longer in active follow-up. Manual extraction from hospital notes can provide supplementary information beyond that available in the HES discharge summary and was used to sub-type some strokes. However, the process was labour intensive. Multiple sources are needed to capture maximum information on stroke events but increasingly with hospitalisation in the acute phase of stroke, HES has an important role. Further development of HES is required to assure validity and coverage.

## Background

Identifying determinants of incident non-fatal stroke is important and longitudinal cohort studies have a vital role to play, alongside clinical registers. Unlike clinical evaluation, where the acute stroke event can be confirmed by clinical signs and diagnostic workup, case ascertainment in epidemiological studies faces several challenges. First, such studies often rely on a self-administered screening questionnaire designed to have high sensitivity and low specificity. Rates of false-positive self-reports of stroke in population studies vary between 25 and 37% [1-4]. Under-reporting has also been observed, with a false-negative rate of 34% for a single question about prior stroke and 10.5% for a stroke symptom questionnaire [5]. Second, reliance on self-reported events in cohort studies is prone to incomplete ascertainment due to drop out. Third, verifying when stroke events occurred, and sub-typing events, will generally require additional clinical information that study participants are unable to provide.

Hospital records routinely collected in electronic form, like NHS HES data, on the other hand, have the potential advantages of diagnostic detail and completeness of follow up, including participants who have dropped out of cohort studies. However, for inclusion, the stroke event must have resulted in hospitalisation and the data are vulnerable to accuracy of coding and completeness.

Another key source of verification is to extract information directly from medical records stored in hospital. Whilst these hospital records have the potential to provide accurate information about the stroke event (including date, sub-typing, imaging, degree of damage and treatment), it is labour intensive, costly and involves access issues which vary from hospital to hospital.

The aim of this paper is to compare the information obtained on non-fatal stroke events from different sources among participants in a large UK based cohort study. The primary question concerns the utility of routine electronic hospital records, such as HES, as an accurate source of information on incident non-fatal stroke in research studies in England. The findings will be used to guide future event tracing procedures and to provide some evidence of certainty on self-reported stroke event rates arising from other population-based cohort studies.

## Methods

The Whitehall II study was established in 1985 as a longitudinal population-based study to examine the socioeconomic gradient in health and disease among 10,308 civil servants (6,895 men and 3,413 women) [6,7]. All civil servants aged 35-55 years in 20 London based departments were invited to participate by letter. In total, 73 per cent of those invited agreed to take part. The baseline examination (Phase 1) took place during 1985-1988, and involved a clinical examination and a self-administered questionnaire. Subsequent phases of data collection have alternated between postal questionnaire alone (phases 2, 4, 6 and 8) and postal questionnaire accompanied by a clinical examination (phases 1, 3, 5, 7 and 9). Home visits were offered at phases 7 and 9 to reduce health-selective attrition bias. The median (and interquartile range) length of follow up from Phase 1 to Phase 9 was 22.4 (17.1 - 23.2) years, with 954 individuals dying during this period. The University College London ethics committee approved the study.

We carried out a validation of self-reported stroke events between Phases 7 and 9 (2002 and 2009) with three external medical sources: (1) visits to hospitals to extract information directly from medical records, (2) linkage to the NHS Hospital Episode Statistics (HES) database, and (3) writing to general practitioners (GPs) for confirmation.

The comparison of information obtained from these sources was limited to the period 2002-09 for two reasons. First, participant consent is needed to access medical records and as consent data would have been over 10 years old for the period before 2002 (phases 1-6), it was decided not to trace self-reported strokes from these earlier questionnaires. Second, the completeness of the HES database was more questionable in earlier years. We also collected information on transient ischaemic attacks, but have not included them in this report as these events are less likely to result in hospitalisation.

### Four sources of stroke notification

(i) Self-report: At data collection phases 7 (n = 6,847), 8 (n = 7,173) and 9 (n = 6,755), the self-administered questionnaire completed by the participants asked whether a doctor had told them they had had a stroke (see Appendix A).

(ii) Hospital Episode Statistics (HES): A link was made to the NHS database, using the participants’ unique NHS identification numbers, for the 10,247 original cohort members (99.4%) for whom the NHS number is known. The study was granted ethical clearance for anonymised electronic linkage with UK health data available for research purposes. HES is a data warehouse containing details (e.g. date, length of stay, diagnoses, procedures) of all admissions to NHS hospitals in England, including acute hospitals, primary care trusts and mental health trusts. HES records also include care provided to NHS patients by the independent sector, including that taking place in treatment centres by the NHS, and care given to private patients in NHS hospitals. The HES database provides reports of participants’ diagnoses on discharge. The following ICD10 codes were selected as primary or secondary diagnoses: I60* (subarachnoid haemorrhage), I61* (intracerebral haemorrhage), I63* (cerebral infarction) and I64*(stroke, not specified).

(iii) Extracting information from hospital records: An attempt was made to visit the hospital to access the participant’s medical records if they had a stroke event identified from self-report information (i) or from HES (ii). Hospital visits were only possible when the hospital name was known (from self-reported information or from HES) and consent to access paper medical records had been obtained at phase 7 or more currently. The Whitehall II study was adopted by the National Institute for Health Research Stroke Research Network (SRN) and the hospital notes were extracted by a nurse from the SRN if the hospital was covered by this network (70 hospitals). If the hospital was not covered by the SRN or if the participant was treated at a private hospital, then a UCL nurse (BM) visited the hospital (31 hospitals). Ethics approval required that we notify the R&D Department at each NHS Trust we were going to visit and acquire a research passport, or letter of access. Information from medical records was extracted onto standard forms for classifying suspected events according to protocol. An endpoint committee was formed to verify diagnosis and to sub-type stroke events into major pathological (ischaemic stroke, intracranial haemorrhage, subarachnoid haemorrhage) and, if sufficient information were available, aetiological stroke sub-types.

(iv) Information from General Practitioners (GP): Letters were sent to participants’ GPs if hospital visits were not possible, for example because no hospital name was given in the self-reported data or there was no electronic record in HES. GPs were also contacted if access to the hospital was denied, medical notes were not available or had been destroyed, or if the hospital was in Wales or Scotland (and if not covered by SRN). In all cases, GPs were only contacted if the participant was alive and not withdrawn from the study, and had given recent consent. GPs were offered £25 to complete a simple questionnaire asking whether their patient had had an ischaemic, haemorrhagic or unspecified stroke, and the date of this event. They were offered an additional £25 per discharge report copied to us. A total of 61 GP letters were sent out and 60 (98.4%) received back, after written and telephone reminders.

## Results

### Validating self-reported strokes

106 stroke events were reported in the self-administered questionnaires among the participants between 2002 and 2009 (Table 1). Of these, 8 (7.5%) were discounted as false positives after looking at the hospital notes or information provided by GPs which suggested that the event was *not* a stroke. A further 4 remain as self-report only as no external evidence was found – these could also be false positives, or stroke events that did not result in hospitalisation and the GP did not respond. Sixty-six (62.3%) of the self-reported strokes were validated by HES data, 15.1% were validated by hospital records extraction alone (i.e. they were only found from manually searching the hospital notes and were not recorded in HES) and 11.3% were validated by GP only.

**Table 1:** Details of verification source for 106 self-reported strokes between 2002 and 2009

***Source of verification***	***Number***	***Percent***
Not verified – external evidence not stroke	8	7.5%
Not verified – no external confirmation	4	3.8%
HES and hospital notes	55	51.9%
HES only	11	10.4%
Hospital notes only	16	15.1%
GP only	12	11.3%
	**106**	**100.0%**

### Additional information provided by HES

A further 47 events coded as strokes were identified among the original baseline participants during 2002 and 2009 in hospitals in England (Table 2). 43 of these participants were no longer active in the relevant data collection phases and 4 had completed questionnaire information but not reported a stroke event. Added to the self-reported strokes, these new 47 stroke events brought the total number of identified strokes to 153. In total, HES provided information on 113 out of 153 stroke events (73.9%). There were 16 stroke events that were found from manually searching the hospital notes which were not recorded in HES.

**Table 2:** Details of source and type of all 153 strokes (self-reported and found in HES) identified between 2002 and 2009

***Source of stroke information***	***Number***	***Percent***
Self-report + other sources (see above)	106	67.6%
HES record but no self-report	47	32.4%
	**145**	**100.0%**
*Type of stroke*		
Non-specified	32	20.9%
Ischaemic	6	55.5%
Cerebral infarction	79	
Haemorrhagic	3	23.5%
Primary intracranial haemorrhage	22	
Subarachnoid haemorrhage	11	
	**153**	**100%**

### Sub-type of stroke

More than half were ischaemic (55.5%), nearly a quarter were haemorrhagic, and 20.9% remain as non-specified (Table 2). Manually extracting hospital records allowed subtyping in 13 (54.2%) of the 24 cases where the HES database recorded “Stroke, not specified”. There was agreement when information on sub-type was present in more than one source.

## Discussion

Validating self-reported strokes with information from the NHS HES database was the most efficient method, compared with manual extraction from hospital notes and corresponding with GPs. Using HES data alone we obtained information on 113 out of 153 strokes (73.9%). The hospital notes extraction exercise allowed us to validate an additional 16 self-reported stroke events not present in HES, and writing to the GPs validated a further 12 stroke events that would have remained as only self-reported. Manual extraction from hospital notes can provide supplementary information beyond that available in the HES discharge summary and we were able to sub-type some strokes using this information. However, as the overall number of strokes in our cohort is relatively small, we are unlikely to have sufficient power to run analyses by type of stroke even with further follow-up. The HES database offers the important advantage of providing information on participants who have left the cohort study. Forty-three stroke events were identified this way. However, further development of HES is needed to reassure users of the validity of the HES coding.

A validation study for a sample of incident stroke cases identified from the EPIC-Norfolk population cohort study, occurring between 1993 and 2003, compared hospital case notes with the entries on a regional based hospital record linkage database and death certification [8]. In general there was excellent agreement between the database and hospital case notes: Out of the sample of 250 incident strokes identified by death certification and hospital record linkage, only 13 had no evidence of stroke in hospital records and there were 4 with other diagnoses. However, they did not validate the strokes that were identified by self-report and the study was limited to comparison of medical records from one hospital (The Norfolk and Norwich University Hospital) and a regional (East Norfolk) hospital database.

It was not our primary aim, nor were we able, to measure the completeness of HES coverage. We do not know the “true” number of non-fatal strokes among the Whitehall participants and so were not able to address the sensitivity or specificity of ascertainment. However, it is noteworthy that about one third of self-reported stroke events were not recorded in HES. Of the 106 strokes identified among Whitehall participants between 2002 and 2009, 66 were validated by HES data. It is possible that the remaining 40 are not in HES because they did not result in hospitalisation, or did not occur in England, or, indeed were not actually strokes (as we suspect in 8 cases). Of these 40, 12 were confirmed by GP, 4 were classified as self-reported only and 16 could be validated using information manually extracted from hospital notes. The fact that these 16 records were not in the HES database raises some concern for its coverage and completeness. We are not aware of published literature on the completeness of the HES coverage; however, there have been several studies on the accuracy of discharge diagnoses. The accuracy of discharge diagnoses in HES was reported to be 87% by The Audit Commission report as long ago as 1991-1993. Dixon et al checked the accuracy of coding, and at that time, concluded that the first three characters of ICD-9 codes were more reliable than full clinical codes [9]. A review in 2001 of 12 studies using HES found the mean coding accuracy rates were 92% for diagnostic codes and 69.5% for operation or procedure codes [10]. In a recently updated review the median diagnostic accuracy (routinely collected data sets versus case notes) was 80.3% [11]. Since the 2002 introduction of Payment by Results, accuracy of the primary diagnosis has improved to 96.0%. The authors conclude that current levels of reported accuracy suggest that routinely collected data are sufficiently robust to support their use for research [11]. Accuracy of hospital discharge coding specifically for haemorrhagic stroke is reported to be excellent, in Newcastle hospitals at least [12].

## Conclusions

We conclude that validation of self-reported stroke in this UK based cohort study is satisfactorily achieved with linkage to the HES database and this had the added advantage of providing information on stroke events among cohort members who no longer actively participate in the study. Where resources allow, ideally a multiple sources approach would be used to include extraction from hospital notes and correspondence with GPs. Future value of the HES dataset will depend on continuing endeavours to maximise hospital and clinic participation in the system.

## Appendix A

Phase 7 Questionnaire: Have you ever been told by a doctor you have had a stroke or transient ischaemic attack (mini stroke/TIA)?

Phase 8 Questionnaire: Since 2002 have you been told by a doctor you have had a stroke or transient ischaemic attack (mini stroke/TIA)?

Phase 9 Questionnaire: Since January 2006 have you ever been told by a doctor that you have had a stroke or TIA. If yes, briefly describe symptoms and their duration. Please give month/year, GP/hospital name, doctor/consultant.

## Competing interests

The authors declare that they have no competing interests.

## Authors’ contributions

EB, AB, AR, CW designed and coordinated the study. BM was the research nurse who carried out the hospital visits, wrote to GPs and gained multiple ethical approvals. AS-G, TB, MS were involved with acquisition of data, management and interpretation. AB drafted the manuscript. All authors read and approved the final manuscript.

## Funding

The Whitehall II study is supported by grants from the Medical Research Council (G0902037), British Heart Foundation (RG/07/008/23674), Stroke Association, National Heart Lung and Blood Institute (5RO1 HL036310) and National Institute on Aging (5RO1AG13196 and 5RO1AG034454). AB and MS are funded by British Heart Foundation.

## Pre-publication history

The pre-publication history for this paper can be accessed here:

http://www.biomedcentral.com/1471-2288/12/83/prepub
